# *Anapleus pescheli*, a new endogean species from Italy and Greece with the key to Western Palaearctic species (Coleoptera, Histeridae, Dendrophilinae, Anapleini)

**DOI:** 10.3897/zookeys.1280.187654

**Published:** 2026-05-28

**Authors:** Tomáš Lackner

**Affiliations:** 1 Naturhistorisches Museum Basel, Augustinergasse 2, 4051 Basel, Switzerland Naturhistorisches Museum Basel Basel Switzerland

**Keywords:** Endogean fauna, new species, southern Italy, Greece

## Abstract

*Anapleus pescheli***sp. nov**. (Italy: Basilicata, Campania, Calabria, Sicilia; Greece: Central Greece, Peloponnesus) is herein described and figured and compared to three congeners from the Western Palaearctic Region. A key to species is given. *Anapleus raddei* (Reitter, 1877) is newly reported from Northern Macedonia and *Anapleus jelineki* Olexa, 1982 is new to Azerbaijan.

## Introduction

The tribe Anapleini was established by Olexa (1982) and belongs to the histerid subfamily Dendrophilinae. It contains a single extant genus *Anapleus* Horn, 1873. The 17 described species of this genus are morphologically similar and are distributed mainly in the Holarctic and Indomalayan realms but with two species known from Mexico (Mazur 2011; Vienna 2015). Many taxa, especially from the Oriental realm await description (Lackner unpublished). According to the recent molecular phylogeny of the family (Seung et al. submitted) the tribe includes the basal-most histerids and currently includes an extinct genus *Cretanapleus* Simon-Pražák & Lackner, 2023 and three extinct species described from the early to mid-Cretaceous as well as Middle Miocene Mexican amber (Jiang et al. 2022; Simon-Pražák et al. 2023, 2024, 2025). Jiang et al. (2022) provided an excellent morphological description of the genus *Anapleus*, and reader is referred there for details.

These beetles live in forest and riparian flood debris, leaf litter, and in soil (occasionally rather deep) (Kovarik and Caterino 2016). They are usually collected by actively sifting forest litter or by using passive methods such as pitfall and flight intercept traps and car-nets. Kryzhanovskij and Reichardt (1976) reported findings of *A. raddei* (Reitter, 1877) also in excrements in southern Azerbaijan; this association has not since been corroborated. The North American *Anapleus marginatus* (J.L. LeConte, 1853) has been frequently found in *Neotoma* Say & Ord, 1825 (woodrat) nest piles (M. Caterino pers. comm.). The Mexican *Anapleus wenzeli* Vomero, 1977 was found among fungi on a cave floor (Palacios-Vargas et al. 2015).

In the eastern Mediterranean, there were formerly three described species of *Anapleus*: *A. raddei* described from the Caucasus, but later found also in Romania (Transylvania), Ukraine, southern Russia, Turkey, and Israel (Mazur 2011); *A. jelineki* Olexa, 1982, described from northern Iran; and *A. wewalkai* Olexa, 1982, described from Anatolia (Turkey) and later also reported from Italy (Calabria and Sicily) (Gomy and Vienna 2001), Syria (Tishechkin 2002), Cyprus (Lackner 2003), and Basilicata (Italy) (Gomy and Kanaar 2004). Gomy and Vienna (2001) expressed doubt about the taxonomic identity of their Italian specimens by describing obvious morphological discrepancies between the described Turkish holotype of *A. wewalkai* and their material, and they also illustrated the venter (Fig. 3) and aedeagus of one of the Italian specimens. The aedeagus they illustrated differs from Olexa’s (1982; Figs 9, 10) aedeagal drawings of the holotype of this species. Despite these inconsistencies, Gomy and Vienna concluded that their Italian specimens were indeed *A. wewalkai* and cited possible geographical variation.

Recently, I received a specimen of an *Anapleus* from Mr Rüdiger Peschel (Chemnitz, Germany) from Peloponnesus (Greece), which had been identified as *A. raddei*. I found more material originating from Greece and southern Italy during several visits of European museums and private collections. Prompted by my Italian colleague and friend Fabio Penati (Morbegno, Italy), who pointed out morphological differences of these specimens regarding individuals from eastern Mediterranean, I decided that Greek and Italian *Anapleus* belong to an undescribed species. Results are presented below.

## Materials and methods

Beetles were carefully removed from their mounting cards after relaxation in lukewarm water and photographed with Canon EOS R7, with Canon RF 100/2,8 L USM IS macro lens Raynox DCR 250. Images were stacked using Zerene Stacker software and edited with Adobe Photoshop 2025®. Aedeagi as well as sterna of three known species were redrawn from Olexa (1982) using Adobe Illustrator CS5. The venter of “*Anapleus wewalkai*” from southern Italy depicted by Gomy and Vienna (2001) has likewise been redrawn from that publication using Adobe Illustrator CS5. The aedeagus of *A. pescheli* was drawn freely by hand using binocular stereoscope Motic BA210E and again redrawn using Adobe Illustrator CS5.

Body-part terminology follows that of Ôhara (1994) and Lackner (2010), and the following abbreviations of morphological measurements are used:

**APW** width between anterior angles of pronotum;

**EL** length of elytron along elytral suture;

**EW** maximum width between outer margins of elytra;

**PEL** length between anterior angles of pronotum and apices of elytra;

**PPW** width between posterior angles of pronotum.

Labels of type specimens were recorded verbatim in single quotations; a vertical bar | separates rows within a label and a double vertical bar || separates individual labels. Additional remarks are given in square brackets.

Specimens examined in this study are deposited in the following collections:

**CFP** Fabio Penati’s private collection, Morbegno, Italy;

**CJP** Jan Pražák’s private collection, Hradec Králové, Czech Republic;

**CND** Nicolas Degallier’s private collection, Paris, France;

**CPV** Pierpaolo Vienna’s private collection, Venice, Italy;

**CTLA** Tomáš Lackner private collection (temporarily housed in ZSM);

**MFNB** Museum für Naturkunde, Berlin, Germany (currently without appointed curator);

**MHNG** Muséum d’Histoire Naturelle, Geneva, Switzerland (G. Cuccodoro);

**MSNG** Museo Civico di Storia Naturale di Genova, Genoa, Italy (M. Tavano);

**NMHW** Naturhistorisches Museum Wien, Vienna, Austria (M. Seidel);

**NMPC** Národní Muzeum Praha, Czech Republic (J. Hájek);

**RMNH** Rijksmuseum van Natuurlijke Historie (Naturalis), Leiden, The Netherlands (O. Vorst);

**ZSM** Zoologische Staatssammlung, München, Germany (M. Balke).

## Results

### Anapleus pescheli sp. nov.

Taxon classificationAnimaliaColeopteraHisteridae

1D005EE5-AADB-5B4B-9B68-F23AC6572B38

https://zoobank.org/D4A645E5-BDD8-4735-8752-260815B2F7BE

Figs 1, 2, 3, 5, 6

#### Type locality.

Greece: Peloponnesus, south of Diakopto.

#### Type material examined.

***Holotype***: • ♂, side-mounted on triangular mounting card, genitalia extracted and glued to the same card as specimen, with the following labels: ‘♂ (printed) || **Greece**: Peloponnes, S Diakopto | Straße nach Kalavrita, 38°07'44"– | 10'26"N, 22°11'49"-14'17"E, 50–850 m, Bachtal, Macchie, AK, | 2.IV.2016, leg. M. Schülke (printed) || HOLOTYPE (vertically written) | *Anapleus pescheli* | spec. nov. | Det. T. Lackner 2025' [MFNB]. ***Paratypes*: Greece** • 10 exs., sex undetermined, same data as holotype [6 exs. MFNB, 4 exs. CTLA]. • 1 ♂ + 4 exs., with the following labels: ‘Greece: Arta, Athamánon Óros | Umg. Skoúpa, Autokescher, | 39° 23'14"–24'12"N, 21°00'52" – | 02'18"E, 285–650 m, 18.IV.2018, | leg. M. Schülke [GR18-13]; || red paratype label identical to the holotype label [3 exs. MFNB, 2 exs. CTLA]. • 3 ♂♂+20 exs., with the following labels: ‘Greece: Peloponnes, S Diakopto | Straße nach Kalavrita, 38°08'11"– | 10'26"N, 22°14'00"–17"E, 500 m, Bachtal, Macchie, Autokescher, | 6.IV.2016, leg. M. Schülke (printed); followed by red paratype label identical to the holotype label [15 exs. MFNB, 4 exs. CTLA, 4 exs. CFP]. • 1 ♀, with the following labels: ‘**GR**: Kokouli | 39,873°N, 20,763°E Gr274G | 28.v.1998 700 m | leg. O. Vorst | Gorge, flood refuse’ (light yellow label, printed); || ‘*Anapleus* / wewalkai Olexa | det. O. Vorst 2022' (light yellow label, printed); || red paratype label [RMNH]. 1 ♂, with the following labels: ‘GR, Peloponnes | Vetaika, Bodenf | 2.5.-9.11.2022 | leg. Manfred Egger’ (black-framed label, printed); || ‘Anapleus raddei | (Reitter, 1877) | det. R. Peschel’ (black-framed label, printed); || red paratype label [NMHW, Peschel collection]. • 1 ex., with the following labels: ‘Grèce Epire | près de Anemorrachi | 400 m 2.v.[19]73 | Löbl (printed-written) || *Anapleus* | raddei Reitt. | leg. S. Mazur 1976 (printed-written) || Mus. Ginevre (written); followed by the red paratype label [MHNG]. **Italy** • 1 ♂, with extracted genitalia, glued to the same mounting card as the specimen, with the following labels: ‘♂ (written) || I.Sic.Gibelmanna | S. Lundberg (printed) || *Anapleus* | wewalkai Olexa | Y. Gomy – Det. 2003' (printed-written) [MHNG]. • 1 ♀, with the following labels: ‘**IT**: Felitto, Calore | 40,366°N, 15,250°E It518K | 20.iv.2008 191 m | leg. O. Vorst | Montane river’ (light yellow label, printed); || ‘*Anapleus* / wewalkai Olexa | det. O. Vorst 2022' (light yellow label, printed); || red paratype label [RMNH]. • 1 ♂, 1 ♀ + 1 ex., with the following labels: ‘CAMPANIA – 11.iv.2004 | tra Rofrano e Sanza (SA) | car-net A. Zanetti legit’ (printed); || ‘*Anapleus* | cfr. wewalkai Olexa | det. Fabio Penati, 2005' (black-framed label, printed); followed by red paratype label [CPV]. • 1 ex., labels identical to the preceding specimen [CND]. • 4 ♂♂, 2 ♀♀ + 21 exs., same data as preceding, but MSNG [3 exs. CTLA; 3 exs. CFP]. • 1 ex, ibid, but NMPC. • 1 ♂, genitalia extracted, glued to the same triangular mounting card as the specimen, left mesotarsus broken off, glued to the same mounting card as the specimen, right mid-leg missing, right metatibia missing, with the following labels: ‘♂’ (written); || ‘Calabria (CS) | Grisolia, 700 mt.loc. | Pantanelle, 8/V/94 | Legit. F. Montemurro’ (written); || ‘Anapleus | wewalkai Olexa | Y. Gomy - Det. 1999' (light-green, black-framed printed/written label); followed by red paratype label [CPV].

**Figures 1, 2.**
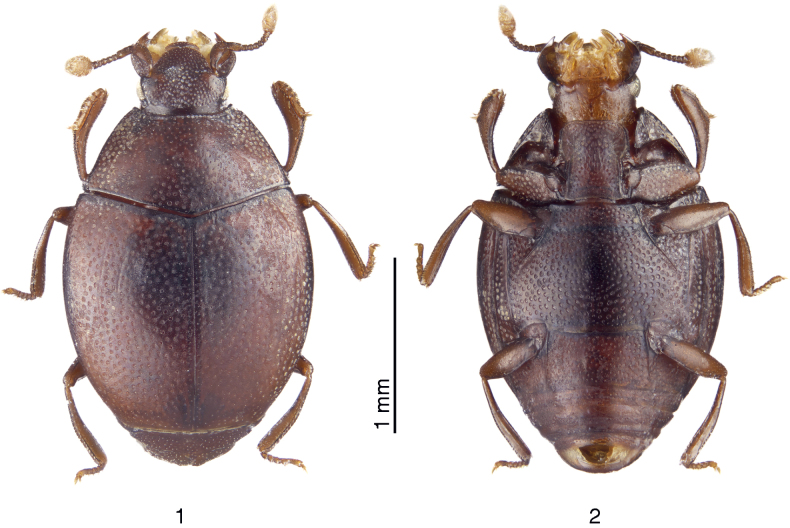
*Anapleus pescheli* sp. nov., holotype. **1**. Dorsal view; **2**. Ditto, ventral view.

#### Diagnostic description.

***Body*** (Figs 1, 2): APW: 0.49–0.56 mm; PPW: 1.14–1.21 mm; EW: 1.34–1.39 mm; EL: 1.22–1.31 mm; PEL: 1.74–1.87 mm. A typical *Anapleus*, colour of pronotum and elytra light to dark brown, covered with rather regular, deep, round punctures separated by 1.0–2.5 times their diameter.

***Head*** densely punctate, punctures largest medially, separated by 0.5× their diameter, supra-orbital area impunctate; frons depressed medially, concave. Epistoma subquadrate, larger than frons, punctation finer and sparser, punctures separated by approximately twice their diameter. Labrum semicircular, minutely punctate, each labral pit with a single seta. Rest of mouthparts not examined. Antennal scape long, approximately four times as long as pedicel; antennomeres III–VII approximately as long as antennal club; intersegmental sutures between antennomeres VIII–XI present.

**Figures 3, 4.**
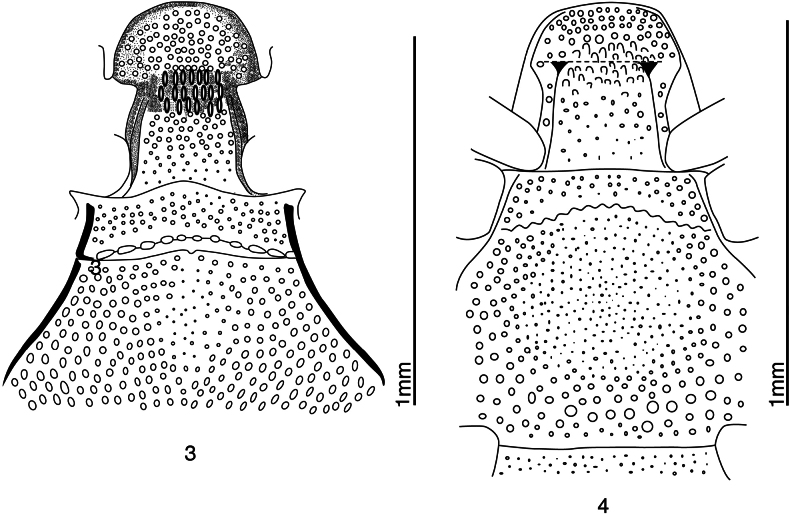
Details of venters of *Anapleus* species. **3**. *A. pescheli* sp. nov., (redrawn from Gomy and Vienna 2001); **4**. *A. wewalkai* Olexa, 1982, holotype (redrawn from Olexa 1982).

***Pronotum*** faint pronotal depressions present; lateral margin almost straight, sides strongly narrowing apically; on basal third lateral margin slightly bisinuate; marginal pronotal stria present, slightly carinate, weakened apically. Pronotal disk laterally with coarse and large punctures becoming very sparse and almost microscopic medially. Pronotal hypomeron glabrous.

***Elytra*** marginal elytral stria complete, slightly carinate; dorsal elytral striae I–II very faint, present on basal elytral half; humeral stria very faintly impressed on basal fourth; other striae missing. Elytral suture slightly elevated. Elytral disk evenly covered with large round deep punctures separated by their approximate diameter, not becoming finer or smaller basally or apically.

***Pygidia*** normal, punctate, punctures separated by about their diameter (propygidium) or sparser (pygidium), apically punctures evanescent.

***Prosternum*** (Figs 2, 3) very broad, prosternal lobe broadly rounded, punctate, process with widely separated subparallel carinal prosternal striae; their apical ends curved inwardly. Prosternal process apico-medially with large, deep elongate punctures.

***Meso-metaventrite*** (Figs 2, 3) typical for the genus; marginal mesoventral stria not discernible; lateral metaventral stria straight, carinate, shortened apically. Intercoxal disk of metaventrite medially strongly convex (in male; in female much less so) here punctures finer than laterally. Meso-metaventral sutural stria undulate, slightly distanced from meso-metaventral suture.

**Figures 5–12.**
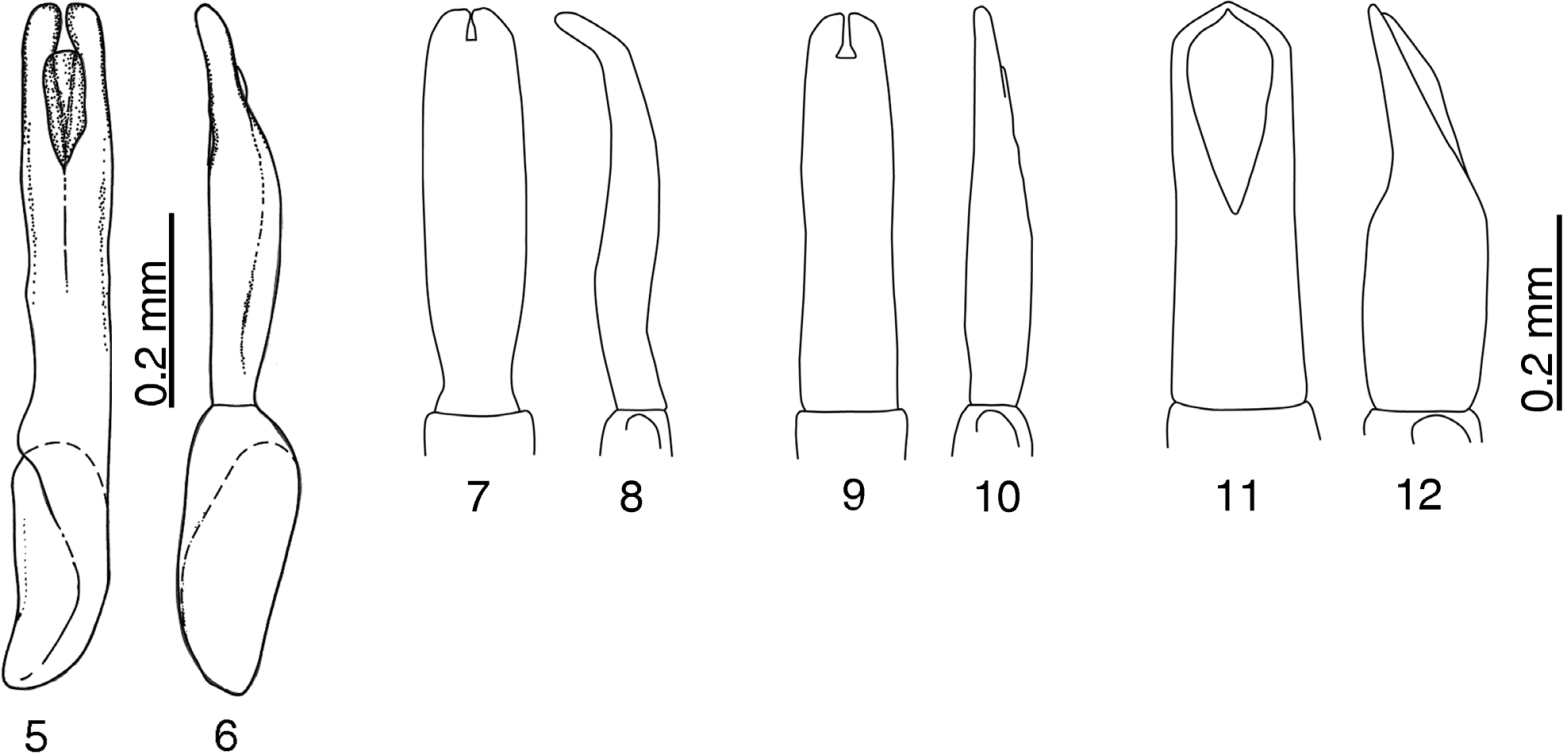
Aedeagi of *Anapleus* species. **5**. *A. pescheli* sp. nov., holotype, ventral view; **6**. Ditto, lateral view; **7**. *A. jelineki* Olexa, 1982, holotype, ventral view (redrawn from Olexa 1982); **8**. Ditto, lateral view; **9**. *A. wewalkai* Olexa, 1982, holotype, ventral view (redrawn from Olexa 1982); **10**. Ditto, lateral view; **11**. *A. raddei* (Reitter, 1877), ventral view; **12**. Ditto, lateral view.

***First visible abdominal sternite*** completely striate laterally; punctuation of disc finer and sparser than that of metaventrite.

***Legs*** unmodified, typical for *Anapleus*.

***Male genitalia***. Aedeagus tube-like, subparallel, parameres “open” on their apical third (Figs 5, 6). Apical third of aedeagus depressed, thinner than the rest (well visible from lateral view).

#### Differential diagnosis.

**Figures 13–18.**
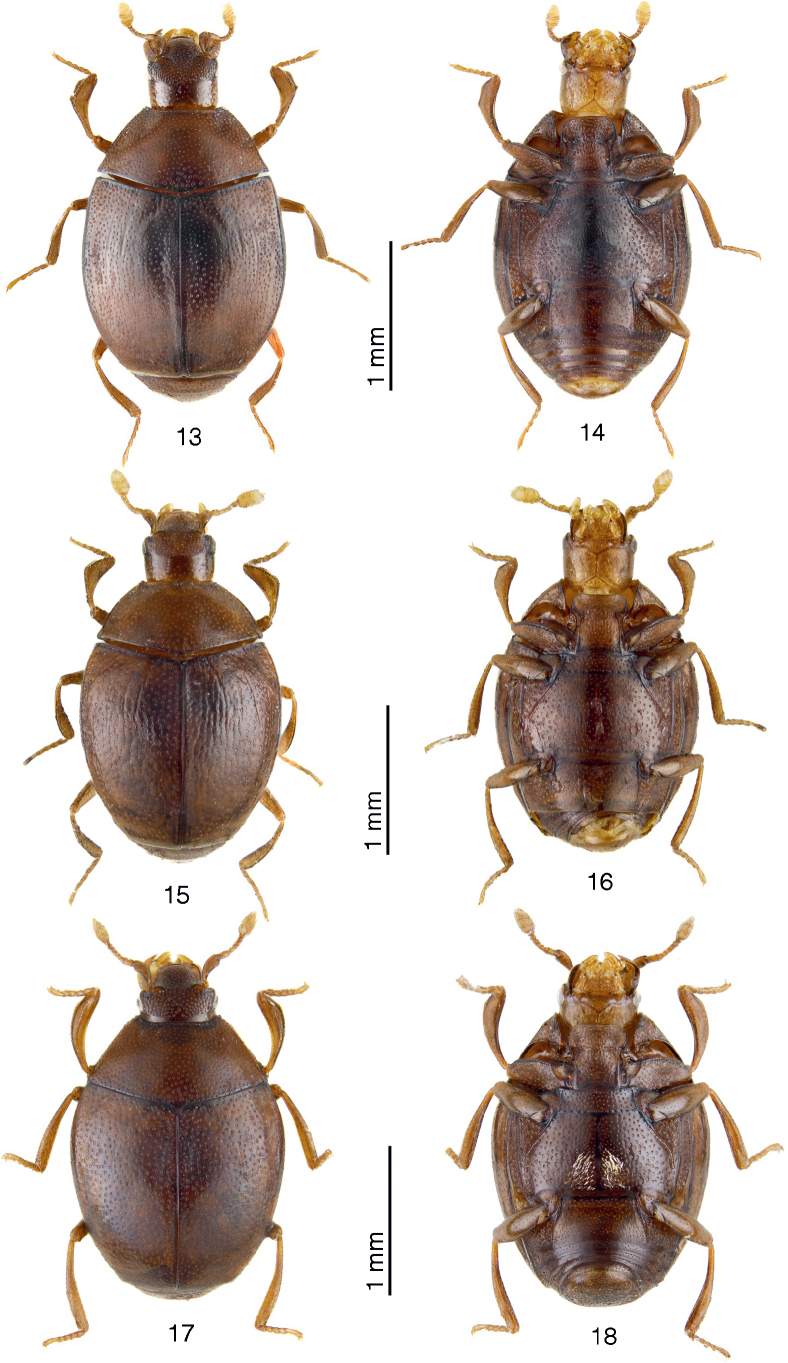
Dorsum and venter of *Anapleus* species. **13, 14**. *A. wewalkai*Olexa 1982, holotype; **15, 16**. *A. raddei* (Reitter, 1877), Romania, Transylvania, Várhegy (= Chinari); **17, 18**. *A. jelineki*Olexa 1982, paratype.

**Figures 19, 20.**
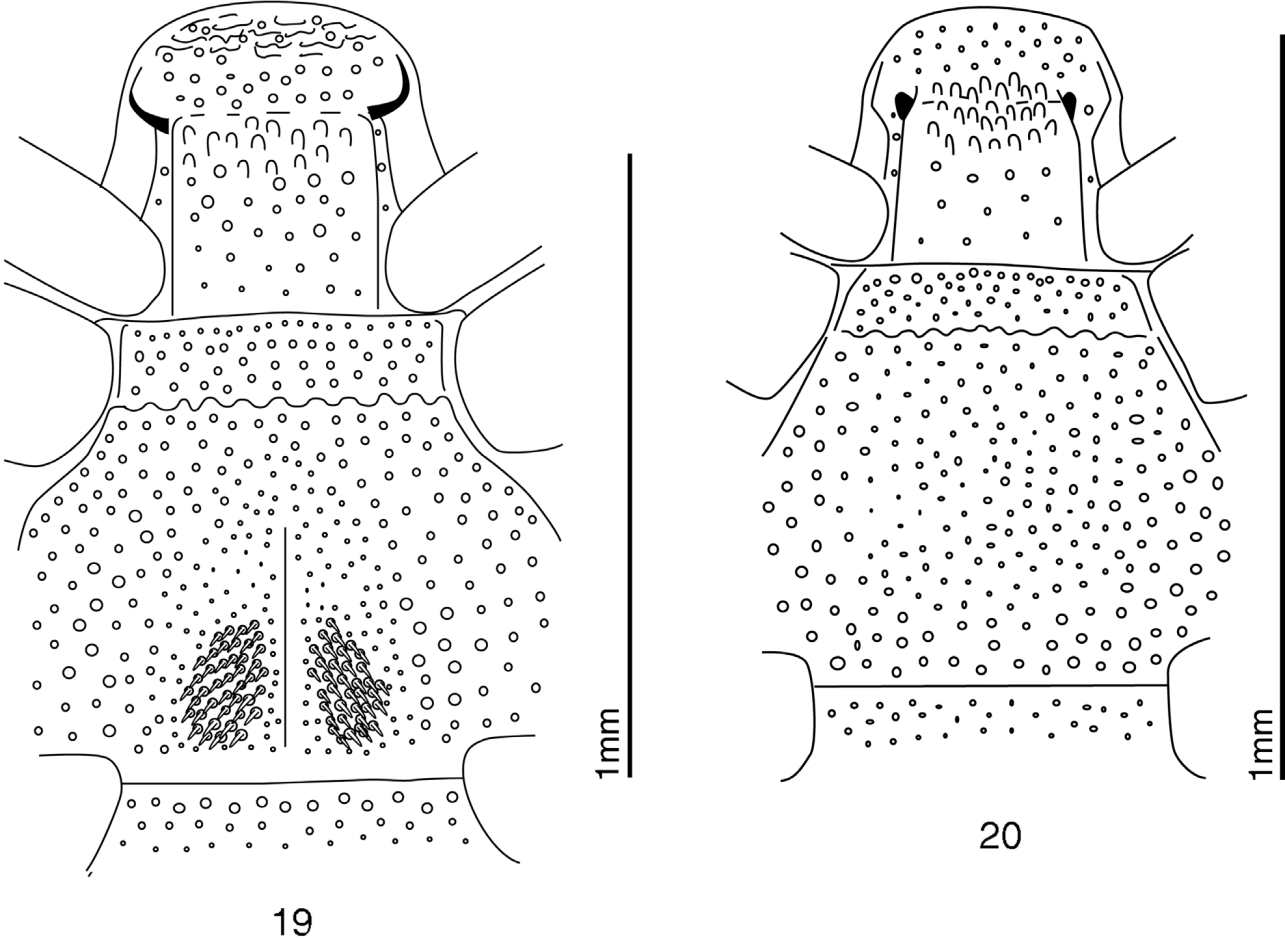
Detail of venters of *Anapleus* species. **19**. *A. jelineki*Olexa 1982, holotype (redrawn from Olexa 1982); **20**. *A. raddei* (Reitter, 1877), Romania, Transylvania, Várhegy (= Chinari) (redrawn from Olexa 1982).

*Anapleus pescheli* sp. nov. has been confused with *A. wewalkai*. It differs from it chiefly by the evenly, densely punctate basal elytral third, especially the area around scutellum (compare Figs 1, 13). Furthermore, the median part of metaventrite of *A. wewalkai* is covered with scattered microscopic punctures (Fig. 4), while that of *A. pescheli* is more coarsely punctuated (Fig. 3). The aedeagi of the two species are different in lateral view. The aedeagus of *A. pescheli* is distinctly convex medially and strongly tapering apically (Fig. 6). The aedeagus of *A. wewalkai* tapers evenly from approximate half-length (Fig. 10) and lacks any median convexity. From two other congeners *A. pescheli* differs as follows: from *A. jelineki* by the darker colouration of the cuticle (compare Figs 1, 17), distanced meso-metaventral sutural stria from the actual meso-metaventral suture (compare Figs 3, 19), but mainly by the absence of depressed setose patch on metaventral base (compare Figs 2, 18; only in male?). The aedeagi of the two species are likewise different: while they are slightly similar from ventral view, aedeagus of *A. jelineki* is distinctly curved from lateral view, while that of *A. pescheli* is not (compare Figs 5, 6 to Figs 7, 8); and, lastly, from *A. raddei*, *A. pescheli* differs by the darker cuticle, even coarser elytral punctation (compare Figs 1, 15), and distanced meso-metaventral stria from the actual meso-metaventral suture (compare Figs 3, 20), but especially by the shape of aedeagus: that of *A. raddei* is rather thick with parameres widely open from approximate mid-length; that of *A. pescheli* is much thinner, with parameres open on their approximate apical third (compare Figs 11, 12 to Figs 5, 6).

#### Remarks.

Olexa’s (1982; redrawn here) illustrations of *Anapleus* aedeagi are somewhat schematic in that he omits the median lobe (duct). While it is not as apparent in *A. jelineki* or *A. wewalkai*, this structure is evident in *A. raddei* and is completely missing in his drawing of this species. It is unknown whether this was done on purpose or whether the median lobe is actually absent from the specimen from Romania on which he based his drawings.

#### Biology.

Unknown. All specimens were collected by sifting forest debris or flood refuse near a montane river; specimens were also collected using car-netting technique.

#### Distribution.

Greece (Central Greece, Peloponnesus); Italy: Basilicata, Campania, Calabria, Sicily.

##### Key to the Western Palaearctic species of *Anapleus*

**Table:** 

1	Basal part of elytra (especially around scutellum) with fine microscopic punctures, separated by several times their diameter (Fig. 13). Metaventrite medially with similar, very fine microscopic punctures (Figs 4, 14). Aedeagus laterally not curved, without median convexity, tapering apically from approximate mid-length (Fig. 10). Distribution: Turkey (Anatolia), Syria, Cyprus	***A. wewalkai* Olexa, 1982**
–	Basal part of elytra (especially around scutellum) with larger round punctures separated by approximately their own to twice their diameter. Punctuation of median part of metaventrite weaker than laterally but punctures never very fine or microscopic	**2**
2	Outer margin of protibia evenly curved, not angulate (Fig. 15). Aedeagus thick, short, parameres separated from their apical mid-length (Fig. 11). Distribution: Georgia, Armenia, Azerbaijan, Russia: Krasnodarskij Kray, Southern Ukraine, Romania, Israel, Iran	***A. raddei* (Reitter, 1877)**
–	Outer margin of protibia angulate (Figs 1, 17). Aedeagus slender, tube-like; parameres either fused almost along their entire length or apically “open” on their apical third at most (Figs 5, 7)	**3**
3	Metaventrite of male with basal depressed sensory patch (Figs 18, 19). Meso-metaventral stria undulate, not distanced from meso-metaventral suture (Fig. 19). Cuticle light brown. Distribution: northern Iran, southernmost Azerbaijan	***A. jelineki* Olexa, 1982**
–	Metaventrite of male without basal depressed sensory patch (Figs 2, 3). Meso-metaventral stria undulate, slightly distanced from meso-metaventral suture (Fig. 3). Distribution: Greece: Central Greece, Peloponnesus, Italy: Campania, Calabria, Sicily	***A. pescheli* sp. nov**.

##### New distributional records

*Anapleus raddei*: Northern Macedonia. 1 ♀, Skopje, 23.vi.1958, C. Besuchet lgt. [MNHG].

*Anapleus jelineki*: Azerbaijan. Astara Region. 9 specs., Talysh Mts, Hirkan NP, Destor env., 38°35'54"N, 48°41'18"E, 850 m, 27.V.2025, Hlaváč, Kocian, Kolimár lgt.; 1 spec., ditto, but 38°35'51"N, 48°40'28"E, 600 m, 29.V.2025, Hlaváč, Kocian, Kolimár lgt.; 1 spec., ditto, but 38°35'49"N, 48°41'15"E, 890 m, 3.VI.2025, Hlaváč, Kocian, Kolimár lgt. [1 ex CPV, 1 ex CTL, 7 exs CJP].

**Remarks**. Olexa (1982) gave NMPC as the type depository of this species. However, the holotype is absent from the NMPC collections (J. Hájek pers. comm. 2025), nor is it present in the collection of late Aldo Olexa (purchased by me in 2004). Its whereabouts are currently unknown.

## Discussion

When Olexa (1982) published his revision of the Palaearctic species of *Anapleus* he stated that beetles of this genus were extremely rare in collections. In fact, *A. jelineki* and *A. wewalkai* were known from only three type specimens and *A. raddei* was known from only six specimens. In the following 40+ years, the situation has improved, and both the number of *Anapleus* specimens and species has substantially increased with proper collecting techniques. Additional records of *A. jelineki* are available from both Iran and southernmost Azerbaijan. It is also possible that Kryzhanovskij and Reichardt’s (1976) records of *A. raddei* from southern Azerbaijan and northern Iran are instead *A. jelineki*. *Anapleus wewalkai* has been recorded from Syria and Cyprus, and *A. raddei* is now known from many specimens that were collected in northern Israel by sifting in early 1980s (Lackner et al. in prep.). Furthermore, the number of described Palaearctic species of *Anapleus* has grown (Mazur 1987; Ôhara 1994). Only the Tajik *Anapleus gracilipes* (Kryzhanovskij, 1966) is currently known only from holotype. Thus, it is not surprising that *A. pescheli* was discovered in the Central Mediterranean region. Discovery of *A. raddei* in Northern Macedonia raises the possibility that both *A. raddei* and *A. pescheli* co-occur there. Using the car-netting technique, several dozen *A. pescheli* have been collected in southern Greece and southern Italy, indicating that the former rarity of this beetle was simply an artefact of it being restricted to poorly sampled endogean habitats. It is likely that the number of records will continue to grow, especially when more leaf-litter and soil sampling is conducted in the Balkans, western Turkey, and countries and territories that lie further to the east.

## Supplementary Material

XML Treatment for Anapleus pescheli

## Additional information

### Artificial Intelligence (AI) use

The author accept full responsibility for the content of the manuscript, including the disclosure of any use of AI.

No AI tools were used in the preparation of this manuscript.

### Author ORCIDs

Т. Lackner https://orcid.org/0000-0002-0108-5785

### Data availability

All of the data that support the findings of this study are available in the main text.
